# Risk factors and medical costs for healthcare-associated carbapenem-resistant *Escherichia coli* infection among hospitalized patients in a Chinese teaching hospital

**DOI:** 10.1186/s12879-016-2176-9

**Published:** 2017-01-17

**Authors:** Xiujuan Meng, Sidi Liu, Juping Duan, Xun Huang, Pengcheng Zhou, Xinrui Xiong, Ruie Gong, Ying Zhang, Yao Liu, Chenchao Fu, Chunhui Li, Anhua Wu

**Affiliations:** Infection Control Centre, Xiangya Hospital of Central South University, Changsha, China

**Keywords:** Healthcare-associated infection, Risk factors, CREC, CSEC

## Abstract

**Background:**

The emergence and spread of Carbapenem-resistant *Escherichia coli* (CREC) is becoming a serious problem in Chinese hospitals, however, the data on this is scarce. Therefore, we investigate the risk factors for healthcare-associated CREC infection and study the incidence, antibiotic resistance and medical costs of CREC infections in our hospital.

**Methods:**

We conducted a retrospective, matched case–control–control, parallel study in a tertiary teaching hospital. Patients admitted between January 2012 and December 2015 were included in this study. For patients with healthcare-associated CREC infection, two matched subject groups were created; one group with healthcare-associated CSEC infection and the other group without infection.

**Results:**

Multivariate conditional logistic regression analysis demonstrated that prior hospital stay (<6 months) (OR:3.96; 95%CI:1.26–12.42), tracheostomy (OR:2.24; 95%CI: 1.14–4.38), central venous catheter insertion (OR: 8.15; 95%CI: 2.31–28.72), carbapenem exposure (OR: 12.02; 95%CI: 1.52–95.4), urinary system disease (OR: 16.69; 95%CI: 3.01–89.76), low hemoglobin (OR: 2.83; 95%CI: 1.46–5.50), and high blood glucose are associated (OR: 7.01; 95%CI: 1.89–26.02) with CREC infection. Total costs (*p* = 0.00), medical examination costs (*p* = 0.00), medical test costs (*p* = 0.00), total drug costs (*p* = 0.00) and ant-infective drug costs (*p* = 0.00) for the CREC group were significantly higher than those for the no infection group. Medical examination costs (*p* = 0.03), total drug costs (*p* = 0.03), and anti-infective drug costs (*p* = 0.01) for the CREC group were significantly higher than for the CSEC group. Mortality in CREC group was significantly higher than the CSEC group (*p* = 0.01) and no infection group (*p* = 0.01).

**Conclusion:**

Many factors were discovered for acquisition of healthcare-associated CREC infection. CREC isolates were resistant to most antibiotics, and had some association with high financial burden and increased mortality.

## Background

Carbapenems have long served as reliable and potent agents against Gram-negative bacilli [1]. Carbapenems are most consistently active against members of the Enterobacteriaceae family [2], however, few treatment options exist for carbapenem-resistant Enterobacteriaceae (CRE) infection, which can result in high mortality [3]. In recent years, carbapenem-resistant *Escherichia coli* (CREC), as one class of CRE, has become a major threat in hospitals worldwide [4–7]. Carbapenem resistance in *E.coli* is an emerging problem that is mainly caused by plasmid-encoded carbapenemases [8–13]. As a result of the emergence of carbapenemases [14], antimicrobial resistance is increasing in most hospitals, and has become a global healthcare problem. CREC strains should be closely monitored because of their potential trend to spread in both hospital and community settings [15].

There are several previous studies on the risk factors for CRE infection [16, 17], but few published studies have specifically evaluated the risk factors for CREC acquisition, especially in China. Therefore, we performed a retrospective study to evaluate the risk factors for healthcare-associated infection (HAI) caused by CREC among in-patients in a teaching hospital in central south China, thus, we could do better in decreasing the incidence of CREC infection.

The case–control–control study design of this study, which utilizes two separate case–control analyses, has become a standard method for the specific identification of risk factors that are uniquely connected to infection by antimicrobial-resistant pathogens [18, 19]. We studied the risk factors for CREC infection through the case–control–control design. In addition, CREC is often resistant to multiple antibiotics; therefore, we investigated the antibiotic resistance and economic burden of CREC infections.

## Methods

### Study design and setting

We conducted a retrospective, parallel, case–control–control study to identify the incidence, risk factors, antibiotic resistance, and medical costs associated with the acquisition of healthcare-associated CREC infection among hospitalized patients treated at Xiangya Hospital, a 3500-bed general hospital in Changsha, Hunan Province, Central South China. The CREC infection group was compared with a no infection group to assess the risk factors for acquisition of CREC infection; meanwhile, the CREC group was compared with the CSEC infection group to evaluate reasons for antibiotic resistance.

Subjects with CREC or CSEC isolated from multiple sites, or on multiple dates, were counted only once, and the data from the first infection was included in the study. Healthcare-acquired CREC or CSEC infection was defined as isolation 48 hours after admission to the hospital. Healthcare-associated infection (HAI) was defined according to the CDC/NHSN surveillance criteria in patients with samples from any specimen source site positive for CR-EC or CS-EC; meanwhile, the patients with CR-EC or CS-EC colonization and community-associated infection (CAI) were ruled out.

### Study population

Patients from whom CREC were isolated from clinical cultures from any source between January 1, 2012 and December 31, 2015 were included in this study. For each CREC patient, we randomly selected two controls from hospitalized patients who were admitted within the same period with CSEC isolated, and the two groups were matched for age and sex. Additionally, we selected two controls from the in-patients admitted within the study period with no bacterial infection, and the two groups were matched for age and sex.

### Microbiological identification and susceptibility testing

An automated broth microdilution method (Vitek 2; bioMérieux, Marcy-l′Étoile, France) was used to perform identification and susceptibility testing. Carbapenem resistance was determined using the disk diffusion method. All isolates with resistance, or intermediate susceptibility to carbapenem were defined as resistant isolates. Clinical and Laboratory Standards Institute document M100-S22 (January 2012) was used for interpretation of the antimicrobial susceptibility testing and ESBL testing, and CREC was defined as *E.coli* resistant to at least one of the carbapenems (imipenem, meropenem, or ertapenem).

Using current EUCAST breakpoints, imipenem MICs of CR-KP isolates ranged from 2 to >32 μg/ml (breakpoint for resistance and intermediate susceptibility MIC ≥ 2 μg/ml); meropenem MICs from 4 to > 32 μg/ml (breakpoint for resistance and intermediate susceptibility MIC ≥ 4 μg/ml); all the isolates had ertapenem MICs in the resistant range (breakpoint for resistance and intermediate susceptibility MIC ≥ 1 μg/ml).

### Data collection

Data were obtained from patients’ medical records, and relative data were recorded on structured abstraction forms. Variables analyzed as possible predictors included demographics (age, sex, marital status, and ward class); clinical departments where strains were isolated; and the history of admission before the infection (within 6 months prior to *E.coli* infection); length of hospital, intensive care unit (ICU) stay before *E.coli* infection; specimen source site (blood, bile, etc.); invasive procedures (urinary catheter insertion, mechanical ventilation, etc.) within 1 month prior to *E.coli* infection; surgical procedures within 1 month prior to *E.coli* infection; administration of drugs (glucocorticoids and immunosuppressive agents), radiotherapy and chemotherapy within 1 month prior to *E.coli* infection; specific co-morbidities included many system diseases (respiratory, central nervous, etc.); exposure (greater than, or equal to, one day) to antimicrobials (cephalosporins, carbapenems, etc.) within 3 months prior to CREC identification.

We also noted any related laboratory results when healthcare-aquired isolation of *E.coli* was recorded in the inspection system, and recorded the drug sensitivity test results obtained from the microbiology laboratory and the economic costs associated with these patients as noted in the financial system. The economic costs included total costs, medical examination costs, medical test costs, total drug costs and anti-infective drug costs.

### Statistical analysis

Continuous variables were presented as mean ± SD, and we used t-tests for comparisons. As the results of the age and average costs of the data for the three groups showed non-normal distribution, they were compared with the median, and the data for two groups were compared using the Wilcoxon rank-sum test. We presented categorical variables as numbers and percentages, and compared percentages using the chi-square test or Fisher’s exact test.

We performed univariate analyses for each of the variables using conditional logistic regression to compare the cases and controls in terms of risk factor analysis. The association between independent variables is shown as the odds ratio with 95% confidence intervals, and variables for which the *P* value was less than 0.05 in the univariate analysis were included in a conditional logistic regression model for multivariate analysis. Multivariate logistic regression models were used to compare each case group and control group. A forward elimination process was used, and adjusted odds ratios and 95% confidence intervals were calculated.

A two-tailed *P* value of less than 0.05 was considered to show statistical significance, and statistical analyses were performed using SPSS 17.0 (SPSS, Inc, Chicago, IL, USA).

## Results

### Incidence of CREC infection

**Fig. 1:**
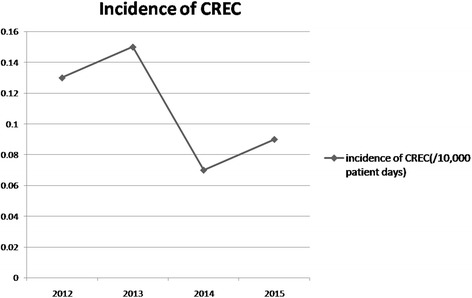
Title: The incidence of carbapenem-resistant *E.coli* (CREC). Legend: The incidence of carbapenem-resistant *E.coli* (CREC) in 2012, 2013, 2014, and 2015 are presented in the figure; we can observe the change of the incidence in the four years from the figure

During the 4-year study period, CREC was isolated from 49 patients who met the criteria for healthcare-associated infection (HAI), including fourteen patients in 2012 (0.13/10,000 patient days), seventeen patients in 2013 (0.15/10,000 patient days), eight patients in 2014 (0.06/10,000 patient days), and ten patients in 2015 (0.10/10,000 patient days). The incidence of CREC infection over the 4-year study presented in Fig. 1.

### Specimen source site and specimen source ward

**Fig. 2:**
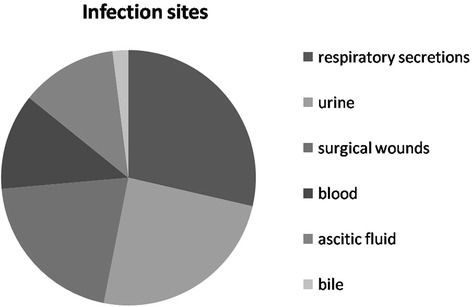
Title: The infection sites of carbapenem-resistant *E.coli* (CREC). Legend: The proportion of carbapenem-resistant *E.coli* (CREC) strains recovered from the sites are presented in the figure, we can observe the regularity of the pathogens distributed

**Fig. 3:**
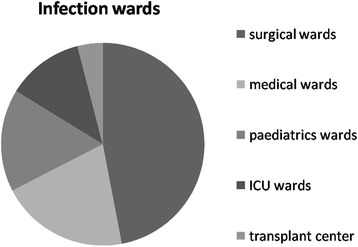
Title: The infection wards of carbapenem-resistant *E.coli* (CREC). Legend: The proportion of carbapenem-resistant *E.coli* (CREC) strains collected in which ward are presented in this figure, we can observe the regularity of the pathogens distributed

A total of 49 patients were included in the case group. CREC was most frequently recovered from respiratory secretions (28.6%), followed by urine (24.5%), surgical wounds (20.4%), blood (12.2%), ascitic fluid (12.2%), and bile (2.0%) (Fig. 2). When a positive culture result was obtained, patients infected with CREC were most frequently staying in surgical wards (46.9%), followed by medical wards (20.4%), pediatric wards (16.3%), ICU wards (12.2%), and the transplant center (4%) (Fig. 3).

### Resistance rate to antibiotics

The antibiotic susceptibility patterns of the isolates from the case and control patients are shown in Table 1. All CREC strains were resistant to ampicillin, ampicillin- sulbactam, cefazolin, ceftriaxone, and cefepime, followed by aztreonam, ceftazidime, ciprofloxacin, levofloxacin, piperacillin/tazobactam, trimethoprim and sulfamethoxazole, cefotetan, cefoperazone/sulbactam, tobramycin, and gentamicin; drug resistance rate to nitrofurantoin and amikacin was relatively low.

**Table 1:** The antibiotic-resistanceof the two groups {carbapenem-resistant *E.coli* (CREC) and carbapenem-susceptible *E.coli* (CSEC)}

	Case (*n* = 49)	Control (*n* = 98)	*p*
Ampicillin	47/47 (100%)	90/96 (94%)	0.08
Piperacillin/tazobactam	38/48 (79%)	10/96 (10%)	0.00
Ampicillin/sulbactam	40/40 (100%)	75/88 (85%)	0.01
Cefoperazone/sulbactam	29/45 (64%)	13/96 (14%)	0.00
Cefazolin	42/42 (100%)	75/92 (82%)	0.00
Ceftazidime	37/39 (95%)	48/87 (55%)	0.00
Ceftriaxone	47/47 (100%)	75/97 (77%)	0.00
Cefepime	49/49 (100%)	50/96 (52%)	0.00
Cefotetan	22/31 (71%)	2/85 (2%)	0.00
Aztreonam	47/49 (96%)	62/97 (54%)	0.00
Tobramycin	26/45 (58%)	51/94 (54%)	0.69
Amikacin	3/48 (6%)	8/98 (8%)	0.68
Gentamicin	26/48 (54%)	49/98 (50%)	0.64
Ciprofloxacin	41/46 (89%)	64/96 (67%)	0.00
Levofloxacin	41/49 (84%)	60/96 (63%)	0.01
Trimethoprim + sulfamethoxaz ole	36/49 (73%)	58/96 (60%)	0.05
Nitrofurantoin	18/44 (41%)	20/98 (20%)	0.01

NOTE. Categorical variables are no/total no (%), case is carbapenem-resistant *E.coli* (CREC), control is carbapenem-susceptible *E.coli* (CSEC)

### Univariate and multivariateanalyses regarding the risk factors of the CREC and CSEC groups

Results of the univariate and multivariate analyses from the comparison of the CREC and CSEC groups regarding the risk factors for healthcare-acquired CREC are shown in Table 2. The univariate conditional logistic regression analysis demonstrated that prior hospital stay (<6 months), urinary catheter insertion, tracheostomy, central venous catheter insertion, gastric tube insertion, urinary system disease, cephalosporins exposure, carbapenems exposure, antifungal agents exposure, glycopeptides and oxazolidinones exposure, low hemoglobin, low blood albumin, and high blood glucose were all risk factors for healthcare-acquired CREC infection. The multivariate conditional logistic regression analysis demonstrated that prior hospital stay (<6 months), incision of trachea, central venous catheter insertion, urinary system disease, low hemoglobin, and high blood glucose were all risk factors for healthcare-acquired CREC infection.

**Table 2:** Univariate and multivariate analyses regarding the risk factors of the carbapenem-resistant *E.coli* (CREC) and carbapenem-susceptible *E.coli* (CSEC) groups

Variable	Study group			Univariate		Multivariable		
	Case (*n* = 49)	Control (*n* = 98)	OR	95% CI	*P*	OR	95%CI	*P*
Demographic characteristics								
Sex, male (%)	20 (41%)	40 (41%)			0.57			
Age {year, median (range)}	51 (0–82)	53 (0–91)			0.69			
Related to hospitalization								
Prior hospital stay (<6 months)	8 (77%)	57 (58%)	2.48	1.10-5.56	0.03	3.96	1.26-12.42	0.02
ICU stay (<6 months)	18 (36%)	22 (22%)	1.48	0.98-2.24	0.06			
Operation history	26 (53%)	28 (29%)	2.53	1.28-5.01	0.01			
Urinary catheter insertion	32 (65%)	59 (60%)	1.55	1.04-2.32	0.03			
Mechanical ventilation	16 (32%)	18 (18%)	1.80	0.97-3.35	0.06			
Tracheostomy	12 (24%)	10 (10%)	1.64	1.09-2.45	0.02	2.24	1.14-4.38	0.02
Bronchofibroscope use	7 (14%)	0 (0%)	72.96	0.53-9980.04	0.09			
Central venous catheter insertion	15 (30%)	7 (7%)	4.48	1.72-11.67	0.00	8.15	2.31-28.72	0.00
Gastric tube insertion	28 (57%)	37 (37%)	1.53	1.09-2.16	0.01			
Wound drainage tube use	18 (36%)	27 (27%)	1.46	0.93-2.29	0.09			
Underlying disorder								
Central nervous diseases	17 (34%)	35 (35%)	0.96	0.47-1.96	0.52			
Respiratory diseases	7 (14%)	18 (18%)	0.74	0.28-1.92	0.35			
Circulatory diseases	11 (22%)	24 (24%)	0.89	0.39-2.01	0.48			
Endocrine diseases	7 (14%)	11 (11%)	1.32	0.48-3.64	0.39			
Hematological diseases	3 (6%)	7 (7%)	0.85	0.21-3.43	0.56			
Digestive system diseases	9 (18%)	23 (23%)	0.73	0.31-1.74	0.31			
Urinary system diseases	11 (36%)	8 (27%)	3.61	1.23-10.61	0.02	16.69	3.01-89.76	0.00
Autoimmune diseases	3 (6%)	5 (5%)	1.21	0.28-5.30	0.54			
Burn	10 (20%)	10 (10%)	2.26	0.87-5.86	0.08			
Antimicrobials agents exposure								
Cephalosporins ^a^	36 (73%)	52 (53%)	2.45	1.16-5.18	0.01			
Carbapenems^b^	19 (38%)	19 (19%)	1.91	1.19-3.04	0.01			
Antifungal agents^c^	17 (35%)	9 (9%)	1.63	1.15-2.32	0.01			
Anti-anaerobic agents^d^	2 (4%)	3 (3%)	1.34	0.22-8.34	0.54			
Glycopeptides^e^ and Oxazolidinones	13 (26%)	10 (10%)	1.73	1.08-2.78	0.02			
Relative laboratory results								
Hemoglobin	104 ± 26	114 ± 26	1.71	1.13-2.59	0.01	2.83	1.46-5.50	0.00
Serum creatinine	116 ± 151	115 ± 25	2.85	0.85-9.60	0.09			
Blood albumin	32 ± 7	36 ± 7	1.65	1.05-2.57	0.03			
Blood glucose	9 ± 7	6 ± 3	2.59	1.16-5.77	0.02	7.01	1.89-26.02	0.00

NOTE. Categorical variables are no/total no (%), and continuous variables are mean ± SD.CI:confidence interval, OR:odds ratio

^a^ Cephalosporins include First, second, third and fourth generation cephalosporins

^b^Carbapenems include imipenem, meropenem, and ertapenem

^c^Antifungal agents include metronidazole and tinidazole

^d^Anti-anaerobic agents include fluconazole, itraconazole, voriconazole and caspofungin

^e^Glycopeptides include vancomycin, teicoplanin, and norvancomycin

### Univariate and multivariate analyses regarding the risk factors in the CREC and no infection groups

The univariate and multivariate analyses results of the CREC and no infection groups are presented in Table 3. The univariate analysis results showed that prior hospital stay, ICU stay, operation history, urinary catheter insertion, mechanical ventilation, tracheostomy, central venous catheter insertion, bronchofiberscope use, gastric tube insertion, wound drainage tube use, urinary system disease, surgical trauma, cephalosporins exposure, carbapenem exposure, antifungal agents exposure, glycopeptides and oxazolidinones exposure, high white blood cell count, low hemoglobin, low blood albumin, and high blood glucose were all risk factors for CREC infection. Multivariate conditional logistic regression analysis demonstrated that, urinary catheter insertion, central venous catheter insertion and carbapenem exposure were all risk factors associated with the acquisition of CREC.

**Table 3:** Univariate and multivariate analyses regarding the risk factors of the carbapenem-resistant *E.coli* (CREC) and no infection groups

Variable	Study group			Univariate		Multivariable		
	Case (*n* = 49)	Control (*n* = 98)	OR	95% CI	*P*	OR	95%CI	*P*
Demographic characteristics								
Sex, male (%)	20 (41%)	37 (38%)			0.43			
Age {year, median (range)}	51 (0–82)	47 (0–82)			0.34			
Related to hospitalization								
Prior hospital stay (<6 months)	38 (78%)	59 (60%)	2.36	1.05-5.30	0.04			
ICU stay (<6 months)	18 (37%)	10 (10%)	3.05	1.60-5.82	0.00			
Operation history	26 (53%)	28 (29%)	2.53	1.28-5.01	0.01			
Urinary catheter insertion	32 (65%)	19 (19%)	5.34	2.58-11.06	0.00	7.14	2.37-21.49	0.00
Mechanical ventilation	16 (33%)	18 (18%)	12.45	2.92-53.05	0.00			
Tracheostomy	12 (24%)	1 (1%)	7.45	1.33-41.65	0.02			
Central venous catheter insertion	15 (31%)	1 (1%)	7.17	2.01-25.60	0.00	8.85	1.04-75.51	0.04
Bronchofibroscope use	7 (14%)	2 (2%)	6.74	1.52-29.83	0.01			
Gastric tube insertion	28 (57%)	4 (4%)	19.25	2.77-133.69	0.00			
Wound drainage tube use	18 (37%)	27 (28%)	3.04	1.60-5.78	0.00			
Underlying disorder								
Central nervous diseases	17 (34%)	23 (23%)	1.73	0.82-3.67	0.11			
Respiratory diseases	7 (14%)	13 (13%)	1.09	0.41-2.63	0.53			
Circulatory diseases	11 (22%)	21 (21%)	1.06	0.46-2.43	0.52			
Endocrine diseases	7 (14%)	8 ( 8%)	1.88	0.64-5.51	0.19			
Hematological diseases	3 (6%)	10 (10%)	0.57	0.15-2.19	0.31			
Digestive system diseases	9 (18%)	16 (16%)	1.15	0.47-2.84	0.46			
Urinary system diseases	9 (18%)	8 ( 28%)	5.06	1.37-18.76	0.02	16.79	0.72-389.5	0.07
Autoimmune diseases	3 (6%)	4 (4%)	1.53	0.33-7.13	0.01			
Burn	10 (20%)	18.54	18.54	2.36-145.58	0.01			
Antimicrobials agents exposure								
Cephalosporins ^a^	36 (73%)	28 (29%)	6.92	3.20-14.97	0.00			
Carbapenems^b^	19 (38%)	4 ( 4%)	7.41	2.46-22.36	0.00	12.02	1.52-95.4	0.01
Antifungal agents^c^	17 (35%)	2 (2%)	4.72	1.65-13.52	0.00			
Anti-anaerobic agents^d^	2 (4%)	3 (3%)	1.35	0.22-8.34	0.54			
Glycopeptides^e^ and Oxazolidinones	13 (27%)	0 (0%)	4.69	1.53-4.31	0.01			
Relative laboratory results								
White blood cellcount	11 ± 7	7 ± 4	1.95	1.11-3.43	0.00			
Hemoglobin	104 ± 26	122 ± 23	2.25	1.41-3.57	0.00			
Blood albumin	32 ± 7	40 ± 5	4.03	2.15-7.58	0.00			
Blood glucose	9 ± 7	5.5 ± 1.8	5.29	2.09-13.41	0.00			

NOTE.Categorical variables are no/total no (%), and continuous variables are mean ± SD.CI:confidence interval, OR:odds ratio

^a^Cephalosporins include First, second, third and fourth generation cephalosporins

^b^Carbapenems include imipenem, meropenem, and ertapenem

^c^Antifungal agents include metronidazole and tinidazole

^d^Anti-anaerobic agents include fluconazole, itraconazole, voriconazole and caspofungin

^e^Glycopeptides include vancomycin, teicoplanin, and norvancomycin

### Medical costs and mortality of the three groups

Comparison of the CREC and CSEC groups, and the CREC and no infection groups, in terms of economic costs are shown in Table 4. Mortality in the CREC group was significantly higher than that in the other two groups. In addition, medical costs of CREC group (including total costs, medical examination costs, medical test costs and total drug costs and anti-infective drug costs) were statistically significantly higher than those for the no infection group. The medical examination costs, and total drug costs and anti-infective drug costs for the CREC group were also statistically significantly higher than those for the CSEC group.

**Table 4:** Economic burden and mortality rate of the three groups

	Case (¥)	control 1 (¥)	control 2 (¥)	*p*1	*p*2
Mortality	6/49 (12%)	1/96 (1%)	1/96 (1%)	0.01	0.01
Total costs	78,900	64,078	17,551	0.05	0.00
examination costs	2923	2571	1062	0.59	0.00
Medical test costs	6329	4649	1389	0.03	0.00
Total drug costs	42,586	29,051	6560.5	0.03	0.00
Anti-infective drug costs	8907	4820	122	0.01	0.00

NOTE. Categorical variables are no/total no (%), continuous variables are median, case is carbapenem-resistant *E.coli* (CREC) , control 1 indicates the carbapenem-susceptible *E.coli* (CSEC) group, and *p*1 indicates the p values for the comparison between carbapenem-resistant *E.col*i (CREC) and carbapenem-susceptible *E.coli* (CSEC). Control 2 indicates the no infection group, and *p*2 indicates the *p* values for the comparison between carbapenem-resistant *E.coli* (CREC) group and no infection group

## Discussion

To our knowledge, few studies have evaluated the risk factors for the acquisition of CREC infection. Therefore, the aim of our matched case–control–control study was to assess the potential risk factors [20] for the acquisition of CREC in clinical specimens from hospitalized patients and to investigate the incidence, medical costs, and antibiotic resistance of the strains from these infections.

During our study period, the incidence of CREC infection was lower than 1/10,000 patient days; it was likely related to the presence of active antimicrobial stewardship teams in the hospital. Although the incidence of CREC is low in CRE, carbapenem resistance in Escherichia coli is also emerging worldwide; the reasons for the spread of CREC are likely limited infection control and antimicrobial control measures [21].

The CREC strains were resistant to at least three kind of antibiotics, the antibiotic resistance of the CREC group was more severe than that of the CSEC group. Compared with the strains from the CSEC patients, most of those from the CREC patients were resistant to cephalosporins, penicillin, aztreonam, ciprofloxacin, and levofloxacin, but the strains remained relatively susceptible to amikacin and nitrofurantoin. We could not have chosen a better way to treat CREC infections considering the above results and according to individual clinical conditions.

The results of our study show that the CREC group was associated with more expenses than the other two groups, particularly in terms of the medical examination costs, total drug costs, and anti-infective drug costs; thus, it appears that antibiotic resistance associated with a higher financial burden. The result is consistent with the study of Bartsch et al. [22]. In our study, although the mortality of the CREC group was significantly higher than that of the CSEC and no infection groups, mortality was not associated with carbapenem resistance [23].

In our study, the univariate analyses of the two case–control groups found many common risk factors, including prior hospital stay, invasive procedures such as urinary catheter insertion [24], incision of trachea, central venous catheter insertion, and gastric tube insertion, urinary system disease, and antibiotic exposure (cephalosporins, carbapenems, antifungal agents, glycopeptides and oxazolidinones). In addition, our study identified unique risk factors, for example, related laboratory results including low hemoglobin, low blood albumin, and high blood glucose. Multivariate analysis demonstrated a number of risk factors, including prior hospital stay (<6 months), tracheostomy, urinary catheter insertion, central venous catheter insertion, carbapenem exposure, urinary system disease, low hemoglobin, and high blood glucose.

The identification of prior hospital stay as risk factor is not unexpected [25]. The environment plays an important role in the spread of antimicrobial resistance, which is a limitless reservoir of antimicrobial resistance genes [26]. Patients who fulfill the variables of prior hospital stay and long total hospitalization time may have had more opportunities to be exposed to additional antibiotics and to other patients carrying antibiotic-resistant organisms [27]. Our result is in agreement with those of a previous study on antibiotic-resistant organisms, which also found these variables to be risk factors [28]. The results suggest that we need to strengthen the management of antibiotics for long-term inpatients and frequently hospitalized patients.

From these two comparisons, it is not surprising to find that invasive procedures, including urinary catheter insertion [24], incision of trachea, and central venous catheter insertion [29] are risk factors for the acquisition of CREC infection. This emphasizes the importance of safety practice in patient care, especially the management of devices. For example, the aseptic technique in catheter use is important as a strategy for the prevention of CREC infections.

There is a close association between healthcare-associated infection and antibiotic use [30–33], especially carbapenem exposure. Thus, in order to more accurately characterize the antibiotic exposure in our study, we assessed the treatment with antibiotics in the 3 months before infection for the case patients and control patients, in this timeframe for data collection is longer than that of other studies [4]. Our findings are in line with those of a recent study that showed the benefit of short-duration, high-dose antibiotic courses as a method to limit unnecessary antibiotic exposure, thus, reduce the risk of antibiotic resistance [34]. According to the suggestion, treatment with high doses and controlled durations is recommended to limit the risk of infections.

It is interesting that the related laboratory results including low hemoglobin and high blood glucose are risk factors for CREC infection, which is different from other studies. The low hemoglobin and high blood glucose are susceptibility risk factors for infection; therefore, special attention should be paid to patients that meet these criteria. We can closely monitor the infection index of these patients while reducing the exposure to risk factors for infection.

One limitation of our study is that we could not assess the patient-to-patient infection spread, we did not collect isolates for gene molecular epidemiologic analysis, thus, we could not assess if there were any outbreaks during the study period. The second is the small study sample size. Moreover, the financial burden is associated with total cost of patients after isolation of CREC or CSEC, of which the direct cost of CREC or CSEC infection was not considered.

## Conclusion

Our results suggest that healthcare-acquired CREC infection may be related to prior hospital stay, tracheostomy, central venous catheter insertion, carbapenem exposure, and urinary system disease. Further, anemia and high blood glucose are important risk factors for the acquisition of CREC infection. Hospital infection control and the implementation of antimicrobial stewardship practices across the continuum of healthcare settings will hopefully help to curb the emergence and spread of CREC infections.

## Abbreviations

CAI: Community-associated infection.
CRE: Carbapenem-resistant Enterobacteriaceae
CREC: Carbapenem-resistant Escherichia coli
CSEC: Carbapenem-susceptible escherichia coli
HAI: Healthcare-associated infection
ICU: Intensive care unit
